# Some Texas Fever Experiments

**Published:** 1893-01

**Authors:** R. R. Dinwiddie

**Affiliations:** Arkansas Agricultural Experiment Station


### THE JOURNAL
OF
### COMPARATIVE MEDICINE AND VETERINARY ARCHIVES.

VOL. XIV. JANUARY, 1893. No. 1.

### SOME TEXAS FEVER EXPERIMENTS.

By R. R. Dinwiddie.

*Arkansas Agricultural Experiment Station.*

During the past three summers attempts have been made to determine the means or medium by which the virus of this disease is carried by Southern cattle, and by which they are thus enabled when shipped or driven North to cause often widespread and fatal disease in Northern stock, and often in this State in cattle only a few miles removed from their original home.

The conditions under which Texas Fever (or Southern Cattle Plague, as it is more appropriately called) occurs, having been described in previous reports from this Station, it is unnecessary to return to them here.

In 1889 (Second Annual Report, Arkansas Agricultural Experiment Station) it was shown that the grasses and surface soil surrounding the roots of the same taken from pastures which had proved very fatal to Northern cattle, did not produce any bad effects on susceptible stock to which they were fed in a dry condition.

It was also shown by my investigations in the summer of 1890 that the statements as to the constant occurrence of bacteria, the so-called germ of Texas Fever, in the blood and organs of all Southern cattle were incorrect, being evidently based upon results obtained by defective bacteriological technique.

This summer and that of 1891 our tests were confined to the two media which now receive the most scientific support as bearers of the Texas Fever virus, namely, the excretions of Southern cattle and Southern cattle ticks. Pens were constructed for the purpose of testing these substances, some 36 feet by 24 feet, others 24 feet by 12 feet. They were put up on level ground and closely boarded in at the bottom, the lowest board being well sunk in the ground. The pens were separated from each other by a space of 12 feet, and the whole inclosed by another fence at some distance from the pens, in order to avoid the risk of spreading disease among stock outside.

The tests made were of a practical nature, and consisted in placing on the ground in some of these pens the excrement of cattle collected both fresh and partly dried, from pastures and in stables in Southern infected regions, and scattering on the surface of other pens ticks picked from cattle of the infected regions of this and other States. In these pens cattle purchased in this neighborhood were afterwards placed and kept during most of the summer, or until the experiment was ended. A modification of these tests consisted in scattering the cattle manure from the South on the hay, and thus introducing it into the stomach of the experimental animals, and of sprinkling the bodies of other cattle with young ticks hatched in the laboratory from eggs deposited by ticks sent from the South.

In the summer of 1891 four cattle were subjected to such tests; during the past summer the experiments were repeated on ten others.

Only such details of these experiments as are necessary for a correct understanding of their nature, and for an intelligent interpretation of the results obtained, will be given here.

*Tests of Cattle Manure, 1891 and 1892.* August 17, 1891, cattle manure to the amount of about two bushels by measure, collected three days before on fields near Pine Bluff, Ark., was scattered on the surface of pen I. August 28th, a like amount, obtained near Fort Smith, was put in the same pen. This manure was not more than a week old when collected.

August 17, 1891, pen II. received about the same quantity of older cattle manure, also from Pine Bluff, and on September 5th a further consignment of the same material from Little Rock.

August 17th, 1891. On this date two cattle, eighteen months old, were placed, one in each of these pens, and kept there until November 6th, or over ten weeks. Like all the others, they were regularly watered and fed hay and a small amount of grain. No regular record was kept of their temperature, as they never showed the slightest signs of sickness. The first frost occurred October 8th. The cattle improved in condition during their confinement.

These and all other cattle experimented with were susceptible to the virus of Texas Fever, being raised north of the "fever line," which, in this part of the State, is south of the Boston mountains.

1892. Pen I. (which during the winter had been well cleaned out and exposed to the weather) received as in the previous year manure dropped by Southern cattle. This was received from Texarkana and Little Rock June 9th and 11th, and from Fort Smith August 22d.

August 2nd two cattle, aged fifteen and eighteen months, were put into this pen and kept there until September 14th in one case, and October 14th in the other. A record of their temperature, taken usually every alternate day, was kept from August 17th, or fifteen days after their entrance, that being the usual time after first exposure before cattle show signs of sickness on infected pastures in the South. The general appearance of the cattle was observed daily.

In the tables appended to this report will be found the record of temperature for all the cattle experimented with during this summer.

It should be said that while the regular temperature readings in such tests are of the greatest value from a diagnostic point of view, the judgment should be based not on this alone, but also on the other symptoms presented during life and on the changes noticed on examination after death. Temporary rise of rectal temperature up to 104° F. in young cattle especially is not uncommon and does not necessarily indicate disease, but continuance of the temperature at this or a higher figure should be considered as due to some pathological condition. In this climate the average normal summer temperature of cattle under two years of age is about 102° F.

Of these cattle in pen I, that numbered II in tables entered pen August 2d (seven weeks after the introduction of manure). Beginning with August 26, the hay in this pen was sprinkled daily with manure soaked and broken up in water. On August 23d a slight and temporary diarrhœa was noticed with a rectal temperature of 103° F. The calf at no time showed any further indication of sickness. The temperature, as may be seen from the table, varied considerably from day to day, but showed no continued elevation. The highest recorded was 104.5° F. on September 22d; the lowest 101.8° F. on September 1st. It was taken out of pen I.

on October 4th and subjected to further tests in pen II. (See No. 22 in tables).

Calf No. 12, also in pen I., never gave any evidence of ill health while subjected to the same test. Highest temperature 102.9° F. September 16th; lowest 101° F. August 27th. It was moved to pen III. on September 19th.

The result of these tests as to the virulence of Southern cattle manure on Northern cattle was considered negative.

*Tests of Southern Cattle Ticks, as Propagators of Texas Fever.* 1891, August 28th. In pen III. (36 feet by 24 feet) were scattered on ground about two hundred living adult ticks picked from "Nation" cattle at Fort Smith on previous day; also on September 7th about the same number sent from Little Rock. The latter had commenced the deposition of eggs.

August 28th, two cattle, seven and one year old, were introduced into this pen. The older animal remained in it until December, but never became invaded by ticks. Only one tick was seen on it at any time, and that was when taken out. The temperature taken at irregular intervals, was always normal, and the cow did not appear to be affected in any way. The younger animal also apparently remained well and free from ticks up till October 14th, when it was thrown down and a large number of young ticks hatched out in the laboratory from eggs deposited by Southern ticks were sprinkled over its body. On the 24th, or ten days later, it was noticed to be indisposed to eat, and from this date to the middle of November did not thrive as well as before, but no rise of temperature was recorded. A small number of ticks grew to maturity on this calf.

November 6th. Two other calves taken from pen I. and pen II. were exposed to the invasion of ticks in pen III., and on one of them a number of young ticks were sprinkled. Some of these ticks arrived at maturity one month later; on the other no ticks appeared. The weather from the middle of November became very cold, the mercury sinking to 12° F. on the 17th, so that nothing could be expected from such a test. These two calves showed no sickness.

October 12th. In pen IV. a two-year-old heifer was similarly sprinkled on the abdomen with young ticks. A few of these were observed fully grown on this animal November 9th. She was too wild to allow of the temperature being taken, but seemed to remain in good health throughout.

1892. Further tests were made of the action of Southern cattle ticks on Northern cattle in pens II., III., IV. and V.

Pen III. received mature ticks sent from Baton Rouge, La., June 24th; from Agricultural College, Mississippi, June 28th and August 21st, and from Little Rock, Ark., August 9th. There were confined in this pen during the summer for various periods, and entering it at different times, eight cattle. Of these, six developed the symptoms of Texas Fever, none, however, in a fatal form.

The three first cattle tested entered pen III. about August 1st. All of these were found to have a high temperature on the 17th of the same month, which only returned to the normal after from two to three weeks. Two did not show marked sickness. and but for the use of the thermometer, would hardly have been considered out of health. The third showed the disease more plainly. It became weak, refused food and fell off very much in condition. Two of these three cattle were examined after death (killed for examination), and showed lesions indicating a mild attack of Texas Fever.

In the second test, made in the same pen, five cattle were introduced during the first and second weeks of September. These were also sprinkled with young ticks hatched out in the laboratory, and all became considerably infested by these parasites. Four of these became very sick within a month after entering the pen, and about three weeks after sprinkling with young ticks. The temperature in these calves began to rise about a week before they showed any distinct signs of sickness. They became very weak, refused all food, and could not be made to rise without lifting. When on foot they walked with a staggering gait; the head was held near the ground, and the breathing was rapid and panting—in fact, they showed plain symptoms of Texas Fever. The urine in none of these became dark or red-colored. After reaching its height the fever gradually subsided to near the normal, but the calves continued for several weeks in a very unthrifty condition, with poor appetite, languor, and weakness. During this convalescence, the morning temperature would indicate one or two degrees of fever (in one case it fell below the normal), while in the evening there would be a rise of three to four degrees, or more.

The cow which did not show fever—an old and feeble animal, suffering from a malignant tumor at the root of the ear—became enormously infested with ticks and died apparently from pure debility.

One of the calves plainly showing the disease was a three months' old suckling.

Pen IV. In this pen no ticks were put on the ground. A fifteen months' old calf was put into it on August 2d and on the same date it was sprinkled with young ticks, the progeny (hatched in laboratory) of several adult ticks obtained at Texarkana, Ark., early in June. These were not of the same species as the common Southern cattle tick. (Species undetermined). Two days later these young ticks, now several times increased in size, were seen in considerable numbers adhering to the body of the calf, but they all disappeared in a few days. This calf remained well during the test of twenty days.

In pen V. another calf of the same age was sprinkled with the young of Louisiana ticks, and raised a fair crop of these. It suffered from a mild attack of fever, only distinguished by the thermometer.

*A Test of Ticks of the Second Generation.* In order to discover if this now well demonstrated fever begetting or propagating property of Southern cattle ticks was a permanent property of these parasites, when removed from their native home, a test was made of the second generation of young.

Young ticks of the first generation from those sent from Louisiana and Mississippi placed on cattle here during the first week of August, reached their full size about the last of the same month. A number of these were picked off the cattle, put in a suitable place in the laboratory, and in a few days egg deposition commenced. At natural temperature the young were hatched out in four weeks, and on September 29th and October 1st a large number of these were put on the body of a healthy four-year-old cow, which was then confined in pen II., previously unoccupied. The cow became manifestly sick on the 11th; that is, twelve days after being thus treated, and died on the 13th, with all the symptoms of an acute case of Texas Fever. Examination after death showed the typical lesions of this disease.

October 4th an eighteen months' old calf, which had been exposed to cattle manure for two months in pen I., without effect, was put in the same pen II., and similarly sprinkled with young ticks of the second generation from those received from Mississippi. It became sick October 12th, with a temperature reaching 106.7° F., refused food and became very thin, but ultimately recovered.

*Summary.* It is seen from the foregoing experiments that the theory which imputes to the dejections of Southern cattle the blame of conveying disease to Northern stock is not sustained by these practical tests. On the other hand, there is abundant evidence of the very important part which cattle ticks play in the production of this Southern cattle fever.

The credit of first experimentally testing and affirming this connection of cattle ticks with Texas Fever is due to Dr. Kilborne (Rep. Bureau of Animal Industry, 1890). The results of this and later experiments of the same kind by the Bureau of Animal Industry are confirmed by the experiments here recorded, so far as the agency of the tick is concerned.

Without assuming too much from this now established fact, a perusal of the experiments given in this report, taken in conjunction with the life history of the cattle tick, will show that we have here an all-sufficient means of explaining the peculiar features of this disease as it occurs on Northern pastures.

It is shown that the progeny of Southern cattle ticks when they invade Northern or susceptible cattle in summer, almost invariably give rise to Texas Fever in these cattle; an unbiased study of the literature on this subject equally shows that no other means by which this disease, under natural conditions, can be transmitted, has been satisfactorily demonstrated.

That the infection in the cases here recorded was produced by a a process of inoculation of cattle by ticks, and not indirectly by microbic infection of the soil, is indicated by the constancy in the time at which fever appeared after the cattle were sprinkled with ticks, and by the fact that in no cases was any fever noticed without the presence of ticks on the body.

The disease exciting power of ticks is not confined to the first generation of those from the South, but exists with equal virulence in the second generation bred in the North. Cases 21 and 22, in pen II., show that fatal secondary infection by Northern cattle can easily occur in this latitude, and in fact, anywhere in the United States. The time which elapsed between the cases of fever induced by successive generations from the same ticks was fifty days (August 17th to October 9th), the only departure from the natural conditions being that the ticks were put on the cattle instead of the latter being invaded from the ground.

From this we may infer that if a first outbreak of Texas Fever occurs as early as two months before the usual advent of freezing weather in that locality, Northern cattle which have recovered from a mild attack (and the number of these is doubtless much larger than has been hitherto supposed), are likely to be responsible for outbreaks later in the year. They become, if carrying ticks on their bodies, walking centres of infection, just the same as Southern cattle, and there is no doubt, if the history of hundreds of cases of Texas Fever which occur every summer in those States bordering on the proscribed regions were more accurately traced that this secondary infection could be frequently demonstrated. In this way the disease may be carried to places where Southern cattle have never been.

*As to Quarantine.* It is evident from the foregoing that when Southern cattle have found their way to non-infected regions any time before September, quarantine should include both cattle and fields or roadways on which they have been. The cattle should be quarantined for a period of four weeks at least (from the date on which they left infected ground). Unless intended for slaughter within two weeks, quarantine should not exceed six weeks. Fields, roads, etc., should be quarantined till late in the fall. If this is done, the infection is confined to its original limits ; if not, it may be distributed far and wide.

On those cattle which die from acute diseases of Texas Fever produced by ticks, these parasites are often found not larger than the size of a grain of wheat, that is, not more than from two to three weeks old, on their host. I have always observed that these half grown ticks do not leave cattle on the death of the latter, but remain adherent to the skin until they also die Hence, such acute cases cannot spread the disease by the agency of the ticks which are on them.

*Texas Fever in Arkansas.* In the northern counties, deaths from this cause occur by far the most frequently among town cows. Farmers who keep their stock confined on their own farms rarely lose cattle from Texas Fever. This I have found to be the case in most of the outbreaks which have been investigated, and it is also indicated by the correspondence which I have had with those counties, chiefly Cross, Independence and Cleburne, where many cattle have died from this cause during the past summer. The original center of infection in these cases may be from cattle driven from further South, but far more frequently it is to be found along the unfenced railway tracts, especially near the depots where stock cars are often delayed. Later in the fall the infection may be widely distributed from this original center.

In the central and southern counties, Texas Fever occurs in a severe form only in imported cattle, native cattle acquiring immunity by one or more mild attacks early in life. Even suckling calves of three months are not exempt from the disease (see case 35 in tables) but in young stock acute fatal cases are rare. (The only fatal case in our experiments was a four year old ; all the others ranging from three months to two years recovered.) One attack, even of the mildest character, seems to confer immunity for that year at least. (Cases 32 and 42.)

While it is shown that ticks form at least the most important agent in the transmission of Texas Fever in the North, and perhaps the only one, it is not certain that this is the case in the South which may be called the home of the virus of the disease. Whether or not susceptible cattle could be protected on Southern pastures by thoroughly keeping off ticks remains to be tested. Bearing on this question is the fact that in one outbreak of Texas Fever at Fort Smith, occurring in March of this year, no ticks could be found on the bodies of those that died, even by the closest inspection.

For those who desire to grade up their cattle by importing improved breeds, the following rules, some of which are not new, may be suggested:

1. Buy cattle raised in the South, and hence already acclimated.
2. When Northern cattle are imported, let it be late in the fall, and let the cattle be under fifteen months old.
3. In the spring prevent the sudden invasion of these cattle by large numbers of ticks, by the methods already given.

NOTES OF CASES, AUTOPSIES, ETC.

*Table A, Case 31.*—Old cow subjected to same test in previous year without showing disease. Temperature first taken August 17th, at which time there was no sign of sickness, except elevated temperature. A few small ticks and one fully grown seen on body; dullness observed about the 21st, continuing for several days, but appetite continued fair. By September 1st apparently as lively as before. Killed September 24th.

*Autopsy.*—No ticks seen on skin; condition fair; mesentery dotted with patches of old blood extravasations from the size of a dime downwards; spleen slightly enlarged (weight three pounds), not abnormally soft; liver shows on capsule many dark, slightly sunken patches, which, on section, are seen to extend some distance into the substance of the organ. Under the microscope these seem to be broken down liver tissue infiltrated with extravasated red blood corpuscles. All the other organs appear normal. Result positive.

*Case 32.*—Bull calf, sixteen months old. Course of disease much like the last; appetite never lost; became enormously invaded by ticks. After temperature had returned to normal, it was subjected to the same test again. (See case 42, Table B). Result positive.

NOTE.—For part of the specimens and material used in the foregoing experiments this Station is indebted to the courtesy of Mr. I. M. Good and Prof. Tait Butler, Agricultural College, Miss., Prof. W. H. Dalrymple, State University, Baton Rouge, La., and Dr. A. E. James, Little Rock, Ark.

*Case 33.*—This calf, fifteen months old, became invaded by ticks from the soil of pen only. Its temperature corresponded closely with that of 31, but otherwise it showed greater sickness, indicated by refusal of food, weakness, emaciation, etc. It was killed for examination, August 30th.

*Autopsy.*—Many ticks of various sizes on skin; flesh and subcutaneous fat normal; contents of thoracic cavity normal; fourth division of stomach contains some small gravel and sand, mucous membrane of same normal. On outer surface of intestines are visible several large patches of blood extravasation; spleen about one-half larger than normal, soft, but pulp not darkened as in acute cases; liver contains much blood and bile, which flows from cut surface; gall-bladder half full of thick, ropy bile of dark yellow color; left kidney shows most marked abnormality, the fat and connective tissue surrounding it being infiltrated with blood to a great extent, and the kidney itself discolored over part of its surface. Bladder contains a little amber colored urine; lymph nodes in lumbar region much darkened and engorged; in other parts they appear to be unchanged. Result positive.

*Case 41.*—Sprinkled with the young of a species of tick obtained on cattle at Texarkana, Ark. Only a few specimens of these were obtained, and adults were not preserved, as it was not noticed that they belonged to another species until mounts were made of the young. (Larvæ resemble those of *Ixodes albipictus*). These young ticks only adhered to the calf a few days and then disappeared. Calf remained well. After five weeks it was subjected to further test in pen III. (See case 36.) Result negative.

*Case 51.*—This calf, two years old, raised a moderate number of ticks after being sprinkled. It never appeared sick, nor lost flesh nor appetite. The thermometric records leave the case doubtful. It was subjected to a second trial in the same pen without effect. (Ticks put on body were from Mississippi and Louisiana).

*Autopsy.*—October 6th, nothing abnormal was seen except traces of discoloration in the capsule of the kidney. Result uncertain.

*Table B, Case 34.*—Old cow, suckling calf, has a suppurating tumor at base of ear, very thin; case described earlier in report; no autopsy. Result considered negative.

*Case 35.*—Calf, three months old, suckling of 34, became invaded with ticks chiefly from soil, but also artificially; went through a non-fatal attack with great weakness, inappetence, languor, etc., with slow convalescence. No autopsy. Result positive.

*Case 36.*—(Same animal as described under 41, Table A). This second test gave positive results. Course of disease similar to that of 35. No autopsy.

*Case 37.*—Eight months old bull calf ; test and result same as in two previous cases. No autopsy. Result positive.

*Case 38.*—Eighteen months old bull calf. (No. 12, of Table A). After being exposed one month in pen I. (to Southern cattle manure test) it was moved to pen III. and young ticks (Mississippi) put on body. It was killed after having passed through a severe attack of fever with great weakness and loss of condition.

*Autopsy*, October 11th, reveals nearly the same appearances as in No. 33, but the liver was more enlarged and the bile almost tar-like in color and consistency; blood, small in amount, thin, and darker in color than normal, forms firm clot; much blood extravasation and effusion of yellow serous fluid around both kidneys and into their capsules; substance of kidneys and lining membrane of pelvis of same congested.

*Case 42.*—(Same animal as described under 32, Table A.) Second crop of ticks raised on body without occasioning any fever.

*Autopsy*, November 5th, revealed no pathological change except traces of old hemorrhage into the capsules of the kidneys. Result of second test, negative.

*Case 21.*—Four year old cow, subjected to test of second generation of young ticks, went through a typical case of acute Texas Fever ; died October 13th, 8 a. m.

*Autopsy*, October 13th, 9 a. m. Large number of ticks on body, none exceeding in size a grain of rice. On removing the skin large patches of blood extravasation were seen on the left side of neck and over the right gluteal region ; fat of subcuta of a deeper yellow than normal ; superficial inguinal glands much enlarged and blood engorged ; little blood. and that of a darker color than normal, flows from cut vessels ; bowels not opened, on outside appear normal ; rectum empty, mucosa of same of a normal, pinkish red color ; third division of stomach full of ingesta ; fourth division contains a little fluid and its lining membrane is deeply congested ; slight peritoneal effusion. Spleen much enlarged, capsule dark slate color, pulp semi-fluid and dark. Liver greatly enlarged (weight 19 pounds) and engorged with blood and bile. Gall-bladder distended with dark, thick, granular bile. Kidneys enlarged and congested, capsule and pelvic lining membrane shows blood extravasations ; connective tissue, fat, and peritoneum for a considerable distance around kidneys a mass of blood and serous effusion. Mesenteric lymph nodes congested. Bladder distended with dark colored urine. Left lung congested, spongy ; right less altered ; pericardium and endocardium blood injected.

*Case 22.*—(Same animal described under 12, Table A.) After passing through test of cattle manure it was put into pen II., and ticks of second generation from the South put on body. It in due time suffered from a severe but non-fatal attack of Texas Fever. No autopsy.

### APPENDIX

### TABLE A.

*Exposure of Northern Cattle to Southern Cattle Manure and Southern Cattle Ticks.*

| No. of Pen. | I. | | III. | | | IV. | V. |
|---|---|---|---|---|---|---|---|
| Pen. How Treated. | June 9, Manure, Little Rock. June 9, Manure, Texarkana. Aug. 22, Manure Ft. Smith. | | June 24, Ticks, Baton Rouge, La. June 28. Ticks, Agricultural College, Miss. Aug. 23, Ticks, Little Rock, Ark. | | | | |
| No. of Animal. | 11. | 12. | 31 | 32. | 33. | 41. | 51. |
| Entered Pen. | Aug. 2. | Aug. 7. | July 24 | Aug. 2. | Aug. 2 | Aug. 2. | July 31. |
| Sprinkled With Ticks. | | | | Aug. 9. (Mississippi.) | | Aug. 2. (Texarkana.) | Aug 2. (Louisiana.) |
| | Record of Temperatures in Degrees Fahr. (At 9 A. M.) | | | | | | |
| 1892, August 17 | 102.5 | 102.2 | 104.6 | 105.5 | 105.7 | ...... | ...... |
| 18 | 102 | 102 | 105 | 105 | 104.3 | 103.2 | 105.3 |
| 22 | 102 | 101.9 | 103 | 104.5 | 104.4 | 102.4 | 102 |
| 23 | 103 | 101.5 | 105.7 | 104.5 | 105 | 102.8 | 102 |
| 24 | 102.5 | 101.8 | 106.7 | 103.2 | 106.3 | 103 | 101.8 |
| 25 | 103.5 | 102 | 107.4 | 103 | 106.8 | 103 | 104.3 |
| 26 | 102.5 | 101.5 | 106.2 | 102.2 | 106.2 | 102 | 102.5 |
| 27 | 102 8 | 101 | 106 | 103 | 106.1 | ...... | 102.4 |
| 29 | 103.5 | 102.5 | 106.5 | 103.8 | 105 | 103.1 | 102.6 |
| 30 | 102 | 101.8 | 105.6 | 102 | 103.8 | 102 | 101.2 |
| September 1 | 101 8 | 101.5 | 103.2 | 102.1 | ...... | 102 | 101 |
| 3 | 102.8 | 102.2 | 103.5 | 102 | ...... | 102.4 | 101.7 |
| 5 | 102.5 | 101.6 | 103 | 102.1 | ...... | ...... | ...... |
| 7 | 102.8 | 101.2 | ...... | 101.8 | ...... | 101.8 | 101.2 |
| 9 | 103.2 | 102.5 | ...... | 102.9 | ...... | 102.1 | 102 |
| 12 | 102.5 | 101.4 | ...... | ...... | ...... | ...... | ...... |
| 14 | 103.8 | 102.8 | ...... | ...... | ...... | ...... | ...... |
| 16 | 103.4 | 102.9 | ...... | ...... | ...... | ...... | ...... |
| 19 | 102.4 | 101.8 | ...... | ...... | ...... | ...... | ...... |
| 22 | 104.5 | ...... | ...... | ...... | ...... | ...... | ...... |
| 24 | 102.9 | ...... | ...... | ...... | ...... | ...... | ...... |
| 26 | 102.8 | ...... | ...... | ...... | ...... | ...... | ...... |

TABLE B.

*Exposure of Northern Cattle to Southern Cattle Ticks of First and Second Generation.*

| No. of Pen. | III. | | | | | IV. | V. | II. | |
|---|---|---|---|---|---|---|---|---|---|
| Pen. How Treated. | June 24. Ticks from Baton Rouge, La. June 28, Ticks from Agricultural College, Miss. Aug. 23. Ticks from Little Rock, Ark. | | | | | | | Sep. 1. Mature Ticks from calf 32 (2d gen. from Mississippi). | |
| No. of Animal. | 34 | 35 | 36 (41) | 37 | 38 (12) | 42 (32) | 51 | 21 | 22 |
| Entered Pen. | Sep. 5. | Sep. 5. | Sep. 7. | Sep. 12 | Sep. 14 | Sep. 9. | July 31 | Sep. 22 | Oct 4 |
| Sprinkled with Ticks. | Sep. 9, Sep. 13 | Sep. 22 | Sep. 13 Sep. 22 | Sep. 22 | Sep. 22 | Sep. 22 | Sep. 22 | Sep. 29 Oct. 1. | Oct 4 |
| | Record of Temperatures in Degrees Fahr. (At 9 A. M.) | | | | | | | | |
| 1892, Sept. 5.... | 102.6 | .. | ...... | ...... | ...... | ...... | ...... | ...... | ..... |
| 7.... | 101.5 | 101.9 | 101.8 | ..... | ...... | ...... | ...... | ..... | ..... |
| 9 ... | 101.8 | 102.7 | 102.1 | ...... | ..... | 102.9 | 102 | ..... | ..... |
| 12.... | 102 | 101.8 | 102.4 | 102 4 | ..... | 102 8 | 102.1 | ...... | ..... |
| 14.... | 102.6 | 102 | 102 5 | 102.5 | 102.8 | ...... | ...... | .. .. | ..... |
| 16. .. | 102 | 102 | 102.3 | 101.8 | 102.9 | 102 5 | 101.5 | ...... | .... |
| 19.... | 101.5 | 101.8 | 102.1 | 101.8 | 101.8 | 102.2 | 102.1 | ...... | ..... |
| 22 ... | 102 | 101.9 | 101.8 | 102 | 103 | 103.5 | 104.2 | 101.3 | ..... |
| 24 ... | 102 | 104.5 | 102.7 | 101.5 | 103.2 | 102.6 | 101.9 | 101 5 | ..... |
| 26.... | 102.3 | 102.8 | 102.7 | 102.3 | 103.6 | 102.8 | 104 | 101.6 | .... |
| 28.... | 102.5 | 102.1 | 103.3 | 103 | 103.8 | 103 | 102 | 101.8 | ..... |
| 30.... | 102 | 103.6 | 104.4 | 102.2 | 103.1 | 103.2 | 101.8 | 101 3 | .... |
| Oct. 3.... | 99 8 | 104.5 | 104.2 | 106.3 | 104.2 | 101.8 | 102 | 100 4 | ..... |
| 5.... | ...... | 103.6 | 106.2 | 106 8 | 105.7 | 101.4 | 101.8 | 101.2 | 101.7 |
| 7 ... | ..... | 106 | 105.6 | 105.5 | 106 5 | 103.2 | ...... | 101.8 | 10[illegible] |
| 8.... | ...... | 104.2 | 104.5 | 102 | 105.2 | ...... | ... .. | ..... | ..... |
| 9.... | ..... | 105.5 | 105.7 | 101.5 | 101.7 | ...... | ...... | ...... | .... |
| 10 ... | ..... | 104.5 | 103.8 | 101.8 | 99 7 | 101.5 | ...... | 107 | 102.3 |
| 11... | ...... | 103.6 | 103 | 101.8 | 100.9 | 101.3 | ...... | 107.5 | 105 |
| 12... | ...... | 103.2 | 102 | 101 3 | ...... | 102 | ...... | 106 | 105 |
| (5:30 p m)12.... | ..... | ..... | 104.3 | 103 5 | ...... | ... .. | ...... | 103.3 | 106.7 |
| 13... | ...... | 101.8 | 101 5 | 101.5 | ...... | 102.2 | ...... | ...... | 105.6 |
| 15.... | ...... | 102 2 | 102 | 102.5 | ...... | 102 | ..... | ...... | 106.7 |
| 17 ... | ...... | 103 8 | 101.8 | 103 2 | ..... | 97.7 | ...... | ...... | 106 |
| (5:30 p m)17.... | ...... | 104 | 103 | 103 9 | ...... | 101.8 | ...... | .... . | 106.6 |
| 19.... | ...... | 102 | 102 7 | 103.6 | ...... | 102.3 | .. ... | ..... | 105 5 |

